# Relationships among Problematic Smartphone Use, Mathematics Achievement, Teacher–Student Relationships, and Subjective Well-Being: Results from a Large-Scale Survey in China

**DOI:** 10.3390/bs12110454

**Published:** 2022-11-16

**Authors:** Da Zhou, Jinqing Liu, Guizhen Ye, Ting Wang, Xiaogang Xia, Jian Liu

**Affiliations:** 1 Faculty of Education, Northeast Normal University, Changchun 130024, China; 2 School of Education, University of California, Irvine, CA 92697, USA; 3 Shandong Provincial Institute of Education Sciences, Jinan 250002, China; 4 School of Mathematical Sciences, Guizhou Normal University, Guiyang 550001, China; 5 Collaborative Innovation Center of Assessment toward Basic Education Quality, Beijing Normal University, Beijing 100875, China

**Keywords:** problematic smartphone use, academic performance, subjective well-being, teacher–student relationships

## Abstract

This study examined the mediating role of mathematics performance and the moderating role of teacher–student relationships on the effects of problematic smartphone use on students’ subjective well-being. Through probability proportionate to size sampling (PPS), a total of 20,321 fourth graders from a city in central China were invited to complete a paper-based mathematics achievement test and an online questionnaire survey, including demographic information, problematic smartphone use, subjective well-being, and teacher–student relationship scales. The results showed that: after controlling for SES and gender, (1) problematic smartphone use had a direct and negative effect on students’ subjective well-being; (2) mathematics performance partially mediated the effects of problematic smartphone use on students’ subjective well-being; (3) teacher–student relationships moderated the effects of problematic smartphone use on mathematics performance/students’ subjective well-being; (4) with the increase in problematic smartphone use, high teacher–student relationships produced a lower rate of the positive moderating effect than low teacher–student relationships. The implications of this study and suggestions for future research are discussed.

## 1. Introduction

With smartphones, we can make phone and video calls, send texts and photos, play games, listen to music, check the news and weather, interact via social media (e.g., Wechat, Facebook, Twitter), and so on. The diverse functionality of smartphones has become an integral part of everyday life for people of all ages and worldwide [1,2]. The large screen size and mobility of smartphones allow people to access the internet anywhere, anytime [1], which has replaced mobile handsets and, to some extent, laptops and other electronic devices. According to *Statista*, the current smartphone users worldwide are 6.648 billion, 83.72% of the world’s population. China, India, and the United States have the highest number of smartphone users [3]. Statistical data show that approximately 70 percent of Chinese owned smartphones in 2020 [4]. Smartphone users are forecast to grow further by several hundred million in the next few years [3].

Although smartphones have made a big difference to our lives, their overuse has also caused many serious problems related to physical fitness, mental health, sleep quality, attention-deficit disorders, emotional experience, and academic performance [1,5,6,7,8,9,10,11,12]. Thus, when exploring the positive influence of smartphones, understanding how we could effectively regulate the adverse effects of smartphones is also essential.

In this study, we investigate the mechanism of the adverse effects of smartphones on elementary students’ well-being and identify moderating factors for alleviating such unfavorable effects. We used the term “problematic smartphone use” to describe the heavy extent to which students used smartphones. We regarded problematic smartphone use (PSU) as compulsive or obsessive use, which is weaker than smartphone addiction [2,13] and thus is more applicable to the population of elementary students whose smartphone use has been limited to a certain level by adults (e.g., parents and teachers). We conducted the current study during the COVID-19 pandemic because problematic smartphone use and well-being issues may be more evident during this time. Given the impact of COVID-19, students in China had to stay at home more and take online classes, which provided them more opportunities to use smartphones and, therefore, more chances to use them for non-class-related purposes, which is especially true for younger students who often lack self-control. As a result, students were under a more substantial tension in time trade-off between smartphone use and study activities [14,15]. Because overuse of smartphones for non-class-related activities (e.g., playing video games, watching online TV series) may hamper study-related activities (e.g., engaging in solving mathematics problems). PSU often causes academic and emotional issues [5,16,17]. This situation may be more evident in East Asian countries because, in these countries, such as China, academic attainment is especially valued by the system [18], and students’ subjective well-being (SWB) and satisfaction with life are often associated with their academic performance in school [1,10]. In brief, this study argues that with the influence of the pandemic, students have more chances to overuse smartphones, which may negatively impact their academic performance and further affect students’ overall well-being and life satisfaction.

Given that low well-being could impact students’ psychological health and cause serious injury to them [19], verifying the mechanism between PSU, academic performance, and well-being is vital, and identifying the moderating factor for alleviating the adverse effects caused by PSU is essential, especially in the background of East Asian culture. In this study, we explored the relationships between PSU, academic performance, and well-being in the context of China, focusing on the moderating role that teacher–student relationships (TSRs) may play. We choose to focus on TSRs because it is an essential malleable factor that directly affects student school functioning [20]. High-quality TSRs may alleviate some negative effects on student academic performance and emotional experience triggered by inappropriate behavioral engagement (e.g., PSU; Internet addiction) by creating a supportive environment in which children are motivated to engage in the classroom actively and appropriately [21]. In brief, this study aimed to verify the direct effect of PSU on students’ SWB, the mediating role of academic achievement between PSU and students’ SWB, and then explore the moderating role of the TSRs in both paths from PSU to academic performance and from PSU to students’ SWB.

### 1.1. PSU and Students’ Subjective Well-Being

SWB refers to how people experience the quality of their lives, comprising an affective component (e.g., pleasant affect and unpleasant affect) and a cognitive component (e.g., life satisfaction). More and more education systems have started to highlight the importance of centering students’ subjective well-being. For example, students’ SWB has become one of the most critical indicators of schooling quality in China, although the Chinese education system has a strong tradition of valuing academic achievements [22]. Thus, how to improve students’ SWB, especially in the context of Chinese schools, has attracted broad attention from researchers.

With the spread of information technology, smartphones can benefit and burden young people’s lives [10,22,23]. Based on the gratifications theory of media uses, appropriate smartphone use for communication may strengthen social connections with family and friends [24,25,26]. However, excessive smartphone use (or PSU) for entertainment or other purposes may affect people’s SWB [10]. Existing studies explored the link between PSU and students’ SWB and found controversial results [1,12,27,28,29,30]. For example, Lepp et al. [12] investigated the relationship between PSU and SWB among 496 American college students and found that PSU was negatively related to students’ SWB. Li et al. [29] also found a similar result in another sample of 516 American undergraduate students. Parallel to these findings, the negative relationship between PSU and SWB was also found based on a sample of 539 Australian undergraduate students [10]. Furthermore, Tangmunkongvorakul et al. [30] collected data from 800 Thai university students. They conducted a survey exploring the link between excessive smartphone use and students’ SWB and found a similar negative link. A possible explanation for these findings was that the exorbitant smartphone use for non-class-related activities hampered students’ emotional experience, thus decreasing students’ SWB [31]. The above studies reveal that the negative link between PSU and well-being was verified by the samples of undergraduates across multiple countries. However, not all research findings are consistent. For example, Samaha and Hawi [1] investigated 300 American university students and found that the PSU did not predict students’ SWB. A reasonable explanation may be that the link between PSU and students’ SWB was mediated by some other factors [5,16,17]. Given the controversial findings and a dominant focus on the adult populations, it is necessary to enrich the current related research to explore further the direct and indirect link between PSU and students’ SWB among a population of elementary school students. Based on these arguments, Hypothesis 1 is proposed.

**Hypothesis** **1.** *There exists a direct effect of PSU on students’ SWB for a sample of elementary school students.*

### 1.2. The Mediating Role of Academic Achievement

Academic achievement is a curtail indicator of how students learn effectively in a high-performing education system [32], which is a key influential factor in students’ emotional experience in East Asian countries [18]. Meanwhile, students’ academic achievement was also affected by some negative behaviors, such as PSU [2]. Thus, academic achievement could play a mediating role between PSU and SWB.

According to self-determination theory, students’ innate psychological needs (e.g., competence, relatedness, and autonomy) are essential for intrinsic motivation, personal growth, and well-being [33,34]. Given that academic achievement could fulfill one’s need for competence and autonomy, especially in a culture where academic achievement is highly valued, students’ academic performance is hypothesized to be associated with their SWB. Guided by this theoretical assumption, several studies have examined the relationship between academic achievement and SWB in different cultural contexts [33,34,35,36]. Bücker et al. [33] conducted a meta-analysis study that involved 47 related studies on the link between academic performance and SWB from different countries. The result showed that the association between academic performance and SWB was *r* =0.164, 95% CI [0.113, 0.216], indicating a positive correlation. Additionally, Choi et al. [34] analyzed the South Korean data (ages 10 and 12, n = 4705) from the International Survey of Children’s Well-Being and found that academic achievement was positively associated with children’s SWB. These findings revealed that the link between students’ academic performance and SWB was positively correlated in different cultural contexts. However, these studies often focus on general academic achievement and seldom explore the relationship between SWB and achievement in a specific subject such as mathematics, science, or Chinese. We know that it was more likely to have an impact on practice to focus on the link between SWB and students’ academic performance in a specific subject instead of general academic performance. Therefore, it is essential to narrow the scope of academic achievement to a specific subject and explore its influence on students’ SWB to enrich the field.

It is worth noting that mathematics is a science of space and quantity that helps in solving the problems of life needing numeration and calculation and providing opportunities for the intellectual gymnastics of man’s inherent powers [37]. For example, mathematics involves counting, recognizing numbers, performing simple operations, solving real-world problems, and so on. Therefore, mathematics is a subject with higher cognition needs. In addition, mathematics is not only fundamental to other subjects but also plays a vital role in cracking various competitive exams [21]. Thus, compared with other subjects, mathematics is an essential subject in school education and needs to receive focus of attention.

However, some negative behaviors (e.g., PSU) harm students’ mathematics learning [2,19,29]. Most existing studies have found a negative association between PSU and students’ general academic performance, with a dominant focus on undergraduates [5,8,11,14,38]. For example, Kates et al. [11] conducted a meta-analysis on the relationship between PSU and academic performance over 10 years (2008–2017), and 80% of the reviewed articles used a sample of undergraduates. The overall meta-analysis indicated that the average effect of PSU on student outcomes was *r* = −0.162. Similarly, a recent systematic review study on undergraduates’ smartphone use and academic performance conducted by Amez and Baert [5] found a negative association between PSU and academic performance based on empirical results. A few studies extended the population from adults to younger children. For example, Zhou et al. [2] conducted a large-scale survey to explore the link between PSU and elementary students’ mathematics performance and found the same results. Based on the above reviews, we hypothesize a mediating effect of mathematics performance between PSU and SWB also exists among elementary school students. Hypothesis 2 is proposed as follows:

**Hypothesis** **2.** *Mathematics performance mediates between PSU and SWB for a population of elementary school students.*

### 1.3. The Moderating Role of Teacher–Student Relationships

Our above review shows that PSU hurts students’ academic performance [2,5,8] and SWB [10,12,30], which naturally inspires us to ask further how educators could effectively alleviate the adverse effects of PSU, resulting in an exploration of moderating factors. In this study, we considered the moderating role of teacher–student relationships. Teacher–student relationships, a two-way interpersonal link, played critical roles in the proximal and distal systems [39,40]. A high-quality teacher–student relationship is usually characterized by warmth and trust, enhancing students’ feelings of security [21]. A supportive teacher–student relationship can create a relaxed and pleasant environment, which effectively alleviates the negative impact of misbehaviors (e.g., PSU) on students’ emotional experience (e.g., students’ well-being) and academic performance [41,42,43]. This idea is supported by attachment theory [44]. Pianta [45] and other researchers have specifically described the importance of teacher–student relationships based on attachment theory [44], suggesting that positive teacher–student relationships help students develop their emotional experience and sense of safety that serves to enhance engagement in academic performance and serves as a buttress against risk [46].

Guided by the attachment theory, several studies have explored the moderating role of teacher–student relationships on the between external behavior factors and academic performance/emotional experience [21,43,47]. Luo et al. [48] examined the moderating of teacher–student relationships on the between of misbehavior and students’ emotional experience, and the results showed that teachers tended to provide more support and help to their students in the context of such a relationship, which encourages students to increase positive emotional experiences while reducing the function of misbehavior. Olivier et al. [43] conducted a study to examine the moderating of teacher–student relationships between terrible external behaviors and students’ internal experiences and found that high teacher–student relationship quality restrained the external bad behaviors and motivated students’ internal experiences. However, previous studies seldom simultaneously focus on the moderating effect of teacher–student relationships between bad behavior (e.g., problematic smartphone use) and academic performance /emotional experience (e.g., subjective well-being) and compare the size of moderating effects. This study aims to bridge this gap by focusing on the moderating effect of teacher–student relationships between PSU and mathematics performance /students’ SWB and comparing its effect size to enrich the current related research further. Hypothesis 3 is presented as follows.

**Hypothesis** **3.** *TSRs positively moderate the links between PSU and mathematics performance/students’ SWB.*

### 1.4. The Present Study

**Figure 1:**
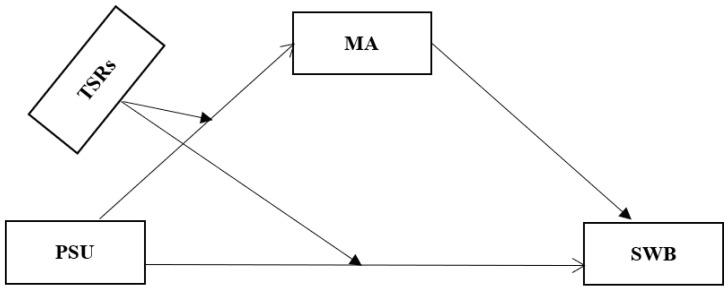
The moderated mediating effects model. Note: PSU = problematic smartphone use; MA = mathematics achievement; SWB = subjective well-being; TSRs = teacher–student relationships.

In summary, this study explores the mediating role of mathematics performance in the effect of PSU on students’ SWB and further explore the moderating role of teacher–student relationships in the effects of PSU on mathematics performance/students’ well-being using path analysis (Figure 1 shows the model) and then examines Hypotheses 1–3.

## 2. Methods

### 2.1. Participants

The Regional Education Assessment Project (REAP), a large-scale assessment project in China, provided data for this study. Through probability proportionate to size sampling (PPS), we sampled a total of 20,321 fourth graders from a city in central China. We first randomly selected about 32 schools from each of the total 10 districts of the city, resulting in a total of 320 schools selected. Then we randomly selected 60~65 students from each school to participate in this study. After excluding the students with missing data, the valid sample size became 19,845 students: 10,231 males (51.6%) and 9613 females (48.4%). Given the current study focuses on students’ use of smartphones, we excluded 8444 students who answered “No” to the question “Do you own your smartphone?” Thus, the sample size of the current study was 11,401 students: 5649 males (49.5%) and 5752 females (50.5%). The ages of these students ranged from 9 to 12 years old, with an average of 10 years old.

### 2.2. Measures

A teacher–student relationship questionnaire, an SWB questionnaire, and a diagnostic questionnaire for PSU were employed in this study. A standardized mathematics achievement test was also used to assess the students’ math proficiency.

#### 2.2.1. Demographic Information

We gathered demographic data on gender and socioeconomic status (SES). Regarding gender, we used “1” to code male and “2” to code female. In terms of SES, we used principal component analysis to determine the composite SES score, a score composed of students’ family possessions, the employment position of the parents, and educational attainment [2,32]. A higher composite score suggests higher SES.

#### 2.2.2. Problematic Smartphone Use

To measure PSU, we used Smartphone Addiction Proneness Scale for Youth [49], a widely used and validated scale in China [2,50,51,52]. This scale comprises 15 items (e.g., *I try cutting my smartphone usage time, but I fail*; *Spending a lot of time on my smartphone has become a habit*). Students were asked to rate the extent to which they agreed with the statements on a four-point Likert scale (1 = strongly disagree to 4 = strongly agree). A higher score suggested a greater likelihood of PSU. The scale was acceptable given its Cronbach’s alpha coefficient was 0.97 and its following model fit indices: $\chi^{2}$ (104, 11,226) = 7392.735, *p* < 0.001, CFI = 0.95, TLI = 0.95, RMSEA = 0.07, and SRMR = 0.03 [53].

#### 2.2.3. Students’ Subjective Well-Being

We used the Index of Well-Being (IWB; [54]) to measure students’ SWB. The IWB is an eight-item scale designed to assess students’ life satisfaction (the first item) and general affect (the remaining items). This scale is self-reported, in which students are asked to rate to which extent they approved the statements on a seven-point Likert scale (1 = not a lot to 7 = a lot). Higher total scores indicate higher levels of SWB. The IWB scale has been adopted in China with satisfactory validity and reliability (e.g., [55]). The model fit indices of the scale were acceptable: $\chi^{2}$ (27, 11,226) = 2815.847, *p* < 0.001, CFI = 0.98, TLI = 0.97, RMSEA = 0.06, and SRMR = 0.02 [53]. The Cronbach’s alpha coefficient was 0.97, which is considered acceptable.

#### 2.2.4. Academic Achievement Test

We used the mathematics achievement test, developed by REAP, to assess students’ academic performance. The test has 27 items, including 10 multiple-choice items and 17 open-response items, each comprising the content domains and the cognition domains (read more details of the test development in [2,21,56]. The mean and standard deviations of the student’s overall scores were 500 and 100, respectively. The internal consistency (Cronbach’s alpha) of the mathematics achievement test was 0.85, which is acceptable because 0.85 > 0.80 [57].

#### 2.2.5. Teacher–Student Relationships

This study adopted the students’ TSRs questionnaire in PISA 2012 to measure students’ perceptions of their relationships with mathematics teachers [40]. The questionnaire consists of five items (e.g., *the math teacher is very fair to me*) in which student reports their agreement from 1 (strongly disagree) to 5 (strongly agree). Higher total scores on the questionnaire indicated more positive perceptions of teacher–student relationships. In addition, the model fit indices of the scale were acceptable: $\chi^{2}$ (5, 11,226) = 598.945, *p* < 0.001, CFI = 0.98, TLI = 0.97, RMSEA = 0.03, and SRMR = 0.02 [53]. The Cronbach’s alpha coefficient of this questionnaire was 0.91 and therefore considered acceptable [57].

### 2.3. Procedure and Analysis

Prior to the study, all students were informed that they had the right to withdraw from the study at any time we introduced it. Students were invited to complete a paper-based mathematics achievement test and an online questionnaire survey. The online survey includes three instruments: PSU, students’ SWB, and TSRs scales. To increase the reliability of the test grading, we trained a group of postgraduate students in mathematics education to grade the test based on the scoring criteria and only allowed them to grade students’ responses after achieving a high scoring consistency (95%). Given the large sample size, we employed a specialist company to enter the test and questionnaire responses for further analysis.

In empirical studies, a lack of careful consideration of common method effects may lead to negative consequences, such as biased estimates of the relationships between constructs employed can affect hypothesis testing. Thus, it is very difficult to make any interpretations of findings when those are influenced by substantive common method effects [58]. Taken together, our analysis started with Harman’s single-factor test to check if serious common method variance existed in this study [59,60]. We then conducted descriptive statistics and correlation analysis to examine the distribution and associations of all variables, which provided adequate evidence for whether we should control covariance variables such as gender and SES [2]. Next, combined with the consideration of research question 2, we used Model 4 in Hayes’s [61] macro program PROCESS to test whether mathematics achievement mediated the relationship between PSU and students’ SWB after controlling for covariance variables. We also computed bias-corrected bootstrap tests with a 95% confidence interval [62] to examine whether the mediation effect was statistically significant. Finally, combined with the consideration of the research question 3, we used Model 8 in the Hayes [61] macro program PROCESS to test the moderated roles of mathematics achievement on the direct path (PSU ➔ students’ SWB) and indirect path (PSU ➔ mathematics achievement), following which we conducted a simple slope analysis to examine how the TSRs moderated the indirect path from PSU to mathematics achievement and the direct path from PSU to SWB. We mainly used SPSS 22.0 macro program PROCESS (International Business Machines Corporation, Armonk, New York, USA) and Mplus 7.0 software (Informer Technologies, Inc., Los Angeles, CA, USA) for data analysis to answer all research questions. The detailed results are as follows.

## 3. Results

### 3.1. Common Method Bias

This study used anonymous responses and reversed scores to control the possible common method bias. Then, the Harman single-factor method was used to test the common method bias [60]. Exploratory factor analysis showed a total of three factors, wherein the interpretation rate of the first factor was 36.59%, which was <40% of the reference value [58], indicating that no serious common method bias existed in this study.

### 3.2. Descriptive Statistics and Correlations

Table 1 presents the results of descriptive statistics and correlation matrices. The mean levels of PSU, mathematical achievement, SWB, and teacher–student relationships were 1.62, 552.82, 5.25, and 4.16, respectively. SES was significantly correlated with all study variables. Specifically, students with higher SES students have significantly stronger SWB (*r* = 0.15, *p* < 0.001), better TSRs (*r* = 0.15, *p* < 0.001), higher mathematics achievements (*r* = 0.34, *p* < 0.001), and lower PSU (*r* = −0.15, *p* < 0.001) than students with lower SES students. Gender was also significantly lower correlated with all study variables. To avoid the effects of gender and SES on the model, the study controlled them in the subsequent mediation effect analysis and moderated mediation effect analysis. In addition, we found that students with higher PSU had significantly lower mathematics achievement (*r* = −0.21, *p* < 0.01) and lower SWB (*r* = −0.21, *p* < 0.01), which laid a foundation for further analysis.

### 3.3. Testing for the Mediation Effect Model

We used Model 4 in Hayes’s [61] macro program PROCESS to test the mediation effects. The results showed that PSU had a significantly predictive effect on students’ SWB (β = −0.19, *t* = −20.08, *p* < 0.001), and the predictive effect is still significant (β = −0.17, *t* = −17.67, *p* < 0.001) after putting the mediation variable of mathematics achievement into the model. Further, PSU has a significant effect on mathematics achievement (β = −0.16, *t* = −18.41, *p* < 0.001), and the predictive effect of mathematics achievement on students’ SWB is also significant (β = 0.13, *t* = 13.44, *p* < 0.001) (see Table 2). Thus, Model 4 used in the study is a partial mediation effect model. It shows that PSU can directly predict students’ SWB and can also predict students’ SWB through the mediating role of mathematics achievement. However, we found that most of the effect comes from the direct effect of PSU on students’ SWB; the mediation effect of the indirect path only represents 10.5% of the total effect. Thus, Hypothesis 2, that the negative relationship between PSU and students’ SWB is mediated by mathematics achievement, was not strongly supported.

### 3.4. Testing for the Moderated Mediation Effect Model

According to the hypothetical mediation effect model, Model 8 in the Hayes [61] macro program PROCESS was used to test the moderated role of mathematics achievement (see Table 3). The results showed that the interaction of TSRs and PSU had significant predictive effect on mathematics achievement (β = −0.02, *t* = −2.94, *p* < 0.01) and students’ SWB (β = −0.02, *t* = −2.54, *p* < 0.05), suggesting the TSRs play a moderating role in the two paths.

**Figure 2:**
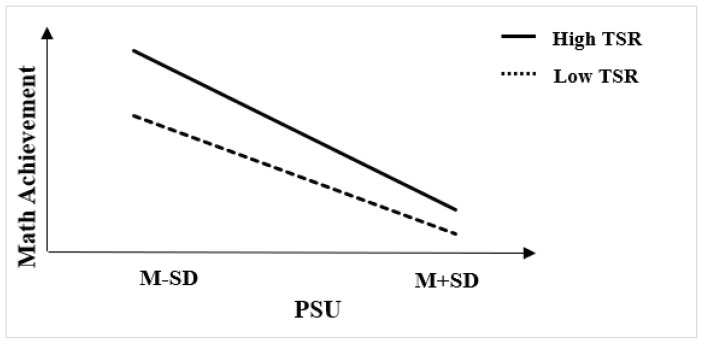
TSRs moderating PSU and math achievement.

Further, we conducted a simple slope test to understand better how the teacher–student relationship moderated the indirect path from PSU to mathematics achievement and the direct path from PSU to SWB. The simple slope test (see Figure 2) indicated that the TSRs could effectively alleviate the negative relationship between PSU and mathematics achievement. However, the slopes in Figure 2 also show that with the increase in PSU, high TSRs produced a lower rate of the positive moderating effect than low TSRs did ($\beta_{high\_TSR}$ = −0.02, 95% CI = −0.03 to −0.02; $\beta_{low\_TSR}$ = −0.01, 95% CI = −0.02 to −0.01). It means that high TSRs could alleviate the negative relationship between PSU and mathematics achievement less effectively when PSU increases, suggesting other ways might be needed to compensate for the decreased moderating effect along with the increase in PSU.

**Figure 3:**
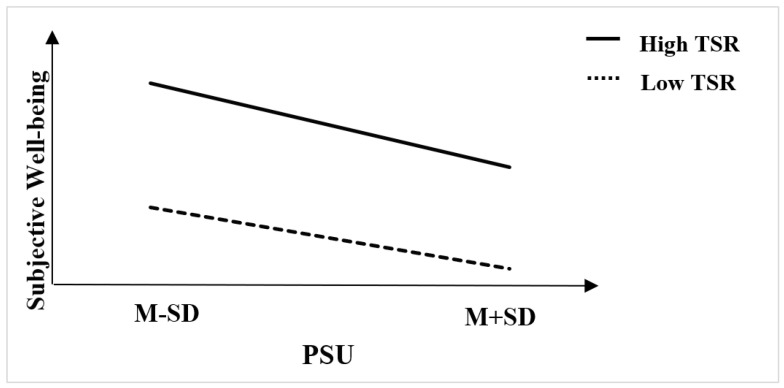
TSRs moderating PSU and SWB.

The results in Figure 3 showed that the teacher–student relationship could effectively alleviate the negative relationship between PSU and students’ SWB. Similarly, high TSRs were not enough to produce a positive moderating effect with the increase in PSU ($\beta_{high\_TSR}$ = −0.15, 95% CI = −0.17 to −0.12; $\beta_{low\_TSR}$ = −0.11, 95% CI = −0.13 to −0.08). It also means that looking for other moderating factors in the education ecosystem was necessary. In brief, Hypothesis 3 was effectively answered.

## 4. Discussion

This study examined the direct effect of PSU on students’ SWB, the mediating effect of mathematics performance between PSU and students’ SWB, and the moderating effects of TSRs between PSU and students’ mathematics performance/SWB using the sample of Chinese elementary students. The results showed that: after controlling for SES and gender, (1) PSU had a direct and adverse effect on students’ SWB; (2) mathematics performance partially mediated the effects of PSU on students’ SWB; (3) TSRs moderated the effects of PSU on mathematics performance/students’ SWB; and (4) with the increase in PSU, high TSRs produced a lower rate of the positive moderating effect than low TSRs.

These results suggest several implications. First, the finding reaffirmed the negative effect of PSU and extended our knowledge of its impact on the population of children in the background of COVID-19. In China, in response to the COVID-19 outbreak, all schools have been closed to minimize the impact of COVID-19 on lives and health [63]. The current pandemic prompts the reform of existing teaching modes in ways that suddenly transited online education from an auxiliary method to the keyway [64]. This change brings obvious problems, including students’ learning engagement and attitudes and so on. Some children were not willing to put down their smartphones after class but used them to do something unrelated to study, which naturally led to students’ PSU. PSU is usually known as phubbing, the act of using a smartphone rather than socializing with others [10], which could cause interpersonal, mental health, psychological problems, and so on and might lead to lower SWB [10,22,65]. For example, Horwood & Anglim [10] conducted a survey to examine the link between PSU and subjective and psychological well-being among Australian adults and found that PSU was correlated with lower well-being on almost all scales. However, these findings are often concluded based on samples of adults. The present study confirmed these findings also apply to younger students by conducting a large-scale survey among Chinese fourth graders, which enriched the related research field. In addition, this study may inspire other researchers to explore how PSU negatively impacts student’ SWB and how to regulate its negative impact in different educational systems and age groups.

Second, different from a dominant focus on students’ general academic performance when researchers studied the mechanisms of how PSU negatively impacted students’ SWB, the present study specified that the mediating role of the achievements of a specific subject, mathematics, which contributes to our understanding. Mathematics, as a subject with high cognition demands, has become one of the subjects of PISA. Montt and Borgonovi [66] explored the link between mathematics achievement and well-being from a cross-cultural perspective and found that students in East Asian countries often experience high levels of stress during their schooling to achieve higher academic performance in mathematics, which naturally leads to a decrease in students’ subjective well-being. However, our finding indicated that mathematics performance partially mediated the effects (just 10.5% of the total effect) of PSU on students’ SWB after controlling for SES and gender, suggesting most of the effect comes from the direct effect of PSU on students’ SWB instead of the indirect effect of mathematics achievement. This is an interesting finding. Given this study was conducted in China, an East Asian country with a long tradition of emphasizing academic achievement, the finding that mathematics achievement plays a mediating role was not surprising [18]. However, the mediation effect only occupied a small proportion of the total effect (about 10%), which might be because our populations are elementary students who might not have internalized the academic-achievement-based culture as a part of their value system. One possible way to test this hypothesis is to see if the mediation effect increases when the students become middle or high school students. Another explanation might relate to China’s education reform in the past decade, which foregrounded students’ psychological feelings and centered students’ mental health education in school education. One potential way to support this argument is to examine a similar population of fourth graders in China every 3 or 4 years and compare the mediating effects. Taken together, the characteristics of East Asian culture may be changing in China.

Third, for further exploring how TSRs moderated the links between PSU and mathematics performance/students’ SWB, Hypothesis 3 was proposed. The results showed that TSRs moderated the effects of PSU on mathematics performance/students’ SWB after controlling for SES and gender, which was congruent with earlier studies (e.g., [41,42,43]). However, interestingly, with the increase in PSU, high TSRs produced a lower rate of the positive moderating effect between PSU and mathematics performance/students’ SWB than low TSRs, indicating when students’ smartphone use became too problematic, high TSRs started to lose its moderating power, suggesting more moderating factors from the education ecosystem should be introduced to reduce the negative impacts of PSU on well-being. According to the *Management Standards for Compulsory Education Schools*, an official document of the Ministry of Education of the People’s Republic of China, the cooperation between schools and families can jointly build a harmonious humanistic environment and promote students’ positive development. Thus, strong parent–child relationships may be one of the moderating factors. According to the attachment theory [40,44], a few studies also showed that a stable family environment is an important contextual factor for children’s growth and development [67,68]. Family can provide emotional warmth and social support for children and thus alleviate the harmful effects of some behavioral factors [68,69]. Some empirical studies provided evidence that parent–child relationships could play a moderating role. For example, Chen et al. [70] found that poorer parent–child relationships related to severe PSU, and Gao et al. [67] found that better parent–child relationships were associated with lower PSU. As such, our study helped to identify the TSRs as a moderating factor; further studies could extend this study by exploring more moderating factors, such as parent–child relationships.

## 5. Limitation and Recommendations

Although this study expands the previous studies by testing a moderated mediation model, this study also has several limitations. First, although we collected large-scale data to study the relationships between the four variables, we could not assess cause and effect relationships directly using cross-sectional data [40]. This is because the cross-sectional study is a single-time analysis of exposure, and the results and causal relationship requires data to be collected from two different time points. As compensation for this limitation, we proposed the causal relations in this study based on the existing studies and related theories. Future studies could use more appropriate methods to explore the cause-and-effect link, such as a longitudinal research design. Second, this study tested the mediating role of mathematics performance in an East Asian culture, a culture that has a long history of valuing, if not extremely, academic achievement, especially mathematics achievement. Thus, mathematics performance may or may not play a different mediating role in other cultures, which requires further validation. Meanwhile, there might be other mediators in the connection between PSU and students’ SWB, which calls for more studies to identify. Third, this research only studied the population of fourth graders. However, there may be differences among other graders. Therefore, testing the model using samples of different graders may help to conclude more general conclusions that apply to a more extensive population range. Fourth, we found that the standardized coefficient for PSU×TSRs was smaller, so we further conducted a simple slope test to know whether a moderating effect exists, and this paper also provided a strong theoretical basis to explain the moderating role of TSRs. However, this may still be not robust, so we will look for a more accurate method to analyze large scale data in the future.

## 6. Conclusions

In sum, this study utilized the data from a large-scale regional assessment of Chinese primary school students to examine series associations among PSU, mathematics performance, SWB, and TSRs, contributing to the field from the following three main aspects. First, the present study contributes by examining the mediating role of mathematics performance and the moderating role of TSRs in the current study model (Figure 1) under an East Asian cultural background. Second, this study extends previous studies by focusing on the achievement of a specific subject, mathematics, rather than the general academic performance, resulting in a better understanding of the impact mechanism. Third, by situating this study within a Chinese education context, this study suggests that culture and educational policy may play roles in determining the impact of PSU on SWB via mathematical achievement. Fourth, this study confirmed the moderating role of TSRs but also showed its limitation, which calls for seeking more moderating factors to support students’ well-being in a smartphone era. In brief, the present study provides very beneficial information and implications on how PSU may cause adverse effects and how it could be alleviated.

## Figures and Tables

**Table 1:** Min, max, mean, standard deviations, and correlations among all the variables.

Variable	Gender	SES	PSU	MA	SWB	TSRs
Gender	1					
SES	0.02 *	1				
PSU	−0.09 **	−0.15 **	1			
MA	−0.03 **	0.34 **	−0.21 **	1		
SWB	0.04 **	0.15 **	−0.21 **	0.19 **	1	
TSRs	0.04 **	0.15 **	−0.24 **	0.13 **	0.24 **	1
Min	1	−1.98	1	200	1	1
Max	2	1.77	4	800	7	5
Mean	1.5	0.26	1.62	552.82	5.25	4.16
SD	0.5	0.66	0.8	84.11	1.95	0.93

Note: PSU = problematic smartphone use; MA = mathematics achievement; SWB = subjective well-being; TSRs = teacher–student relationships; SD = standard deviation. * *p* < 0.05, ** *p* < 0.01.

**Table 2:** Standardized coefficients for mediation effect model.

Predictors	Model 1 (SWB)		Model 2 (MA)		Model 3 (SWB)	
	β	***t***	β	***t***	β	***t***
Gender	0.02	1.98 *	−0.05	−5.37 ***	0.03	2.73 **
SES	0.12	13.19 ***	0.32	35.62 ***	0.08	8.32 ***
PSU	−0.19	−20.08 ***	−0.16	−18.41 ***	−0.17	−17.67 ***
MA					0.13	13.44 ***
$R^{2}$	0.06		0.14		0.07	
*F*	227.53 ***		614.24 ***		218.99 ***	
Indirect effect			B	Boot SE	LLCI	ULCI
MA			−0.02	0.002	−0.03	−0.02

Note: * *p* < 0.05, ** *p* < 0.01, *** *p* <0.001.

**Table 3:** Standardized coefficients for moderated mediation effect model.

Predictors	Model 1 (MA)		Model 2 (SWB)	
	β	***t***	β	***t***
Gender	−0.05	−5.45 ***	0.02	2.38 *
SES	0.31	34.58 ***	0.06	6.35 ***
PSU	−0.16	−17.07 ***	−0.13	−13.58 ***
TSRs	0.05	5.35 ***	0.19	19.82 ***
PSU × TSRs	−0.02	−2.94 **	−0.02	−2.54 *
MA			0.12	12.69 ***
$R^{2}$	0.14		0.10	
*F*	375.49 ***		216.49 ***	

Note: * *p* < 0.05, ** *p* < 0.01, *** *p* <0.001.

## Data Availability

The data is confidential.
